# The effect of deuterium on the photophysical properties of DNA-stabilized silver nanoclusters†

**DOI:** 10.1039/d1sc05079f

**Published:** 2021-11-25

**Authors:** Cecilia Cerretani, Gustav Palm-Henriksen, Mikkel B. Liisberg, Tom Vosch

**Affiliations:** Department of Chemistry, University of Copenhagen Universitetsparken 5 Copenhagen 2100 Denmark cece@chem.ku.dk tom@chem.ku.dk

## Abstract

We investigated the effect of using D_2_O *versus* H_2_O as solvent on the spectroscopic properties of two NIR emissive DNA-stabilized silver nanoclusters (DNA–AgNCs). The two DNA–AgNCs were chosen because they emit in the same energy range as the third overtone of the O–H stretch. Opposite effects on the ns-lived decay were observed for the two DNA–AgNCs. Surprisingly, for one DNA–AgNC, D_2_O shortened the ns decay time and enhanced the amount of µs-lived emission. We hypothesize that the observed effects originate from the differences in the hydrogen bonding strength and vibrational frequencies in the two diverse solvents. For the other DNA–AgNC, D_2_O lengthened the ns decay time and made the fluorescence quantum yield approach unity at 5 °C.

## Abstract 2

We investigated the effect of using D_2_O *versus* H_2_O as solvent on the spectroscopic properties of two NIR emissive DNA-stabilized silver nanoclusters (DNA–AgNCs).

## Graphical abstract

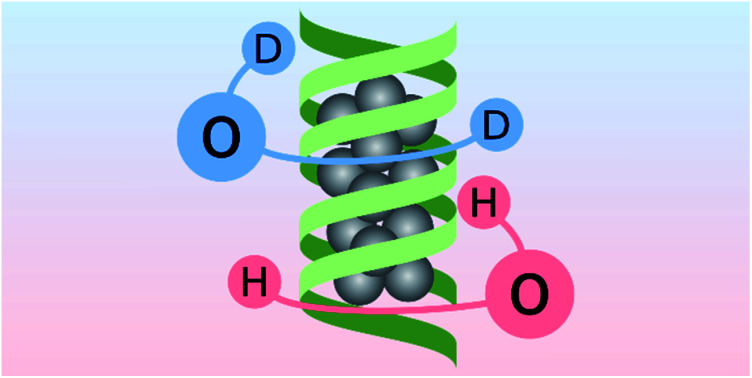

## Introduction

DNA-stabilized silver nanoclusters (DNA–AgNCs) were first introduced by Petty *et al.*^1^ and consist of a limited number of silver atoms and cations embedded in one or more DNA strands. A comprehensive introduction to the structure/property relationship of this class of emitters can be found in a review by Gonzàlez-Rosell *et al.*^2^ Recent findings show that the interplay between the DNA host sequence and the emissive properties of the stabilized silver nanoclusters is intricate and complex. For example, replacing a single guanine with an inosine resulted in a longer fluorescence decay time and a quantum yield (*Q*) increase from 0.25 to 0.63.^3,4^ This is remarkable since the only difference between these two nucleobases is a single amino group, which seems to control the amount of non-radiative decay.^4^ Inspired by these results, we decided to evaluate the effect of exchanging H_2_O with D_2_O on the photophysical properties of DNA–AgNCs. It is well-known that substituting H_2_O with D_2_O lengthens the excited state decay time and increases the luminescence quantum yield of both ns-lived fluorescence from organic fluorophores,^5,6^ as well as µs- and ms-lived emission from trivalent lanthanide ions.^7,8^ The main reason for this is the lower vibrational frequency of the O–D stretch with respect to the O–H stretch, resulting in less solvent-mediated non-radiative decay.^5,9^ Maillard *et al.* showed that H_2_O and alcohols can act as weak quenchers and proposed a model of resonant energy transfer from the electronic excited state to vibrational overtones of the O–H bond.^5^ Previous studies on a green emissive DNA–AgNC reported no significant effect of exchanging H_2_O with D_2_O on the ns-lived excited state decay.^3,10^ To assess the effect of D_2_O as a solvent, we chose two well-characterized DNA–AgNCs that emit close to 750 nm, which is where the absorption bands of the third overtone of the antisymmetric, symmetric O–H stretch and combination bands thereof are located.^5^ Both DNA–AgNCs are stabilized by multiple DNA decamers. One DNA–AgNC contains 16 Ag atoms embedded in two 5′-CACCTAGCGA-3′ strands (further defined as DNA–Ag_16_NC),^11,12^ and its structure can be found in the PDB database (6JR4). The structure of the second DNA–AgNC has not been determined yet, but the hydrodynamic volume^13^ suggests it is likely wrapped in two or three 5′-CCCGGAGAAG-3′ strands (further referred to as DNA721–AgNC). At 25 °C in a 10 mM ammonium acetate (NH_4_OAc) aqueous solution, DNA–Ag_16_NC has a moderate *Q* of 0.26 and a decay time that is very temperature-dependent.^12^ On the other hand, DNA721–AgNC is characterized by a high *Q* of 0.73 and a decay time that is largely independent of temperature.^13^

Surprisingly, and against all expectations, the ns decay time of DNA–Ag_16_NC was found to be shorter and *Q* lower upon using D_2_O as solvent *versus* H_2_O. Additionally, D_2_O enhanced the red-shifted µs-lived emission, which was negligible in H_2_O. Dual emissive DNA–AgNCs, featuring both ns- and µs-lived emission, have been only recently reported in literature,^14,15^ and it was surprising to discover µs-lived emission for this well-characterized DNA–Ag_16_NC upon addition of D_2_O. For DNA721–AgNC, a more expected behavior was observed using D_2_O as a solvent: the ns-lived decay lengthened and *Q* increased, reaching unity at 5 °C. To the best of our knowledge this is the highest reported *Q* value for a NIR-emitting DNA–AgNC.

## Results and discussion

### DNA–Ag_16_NC

**Fig. 1:**
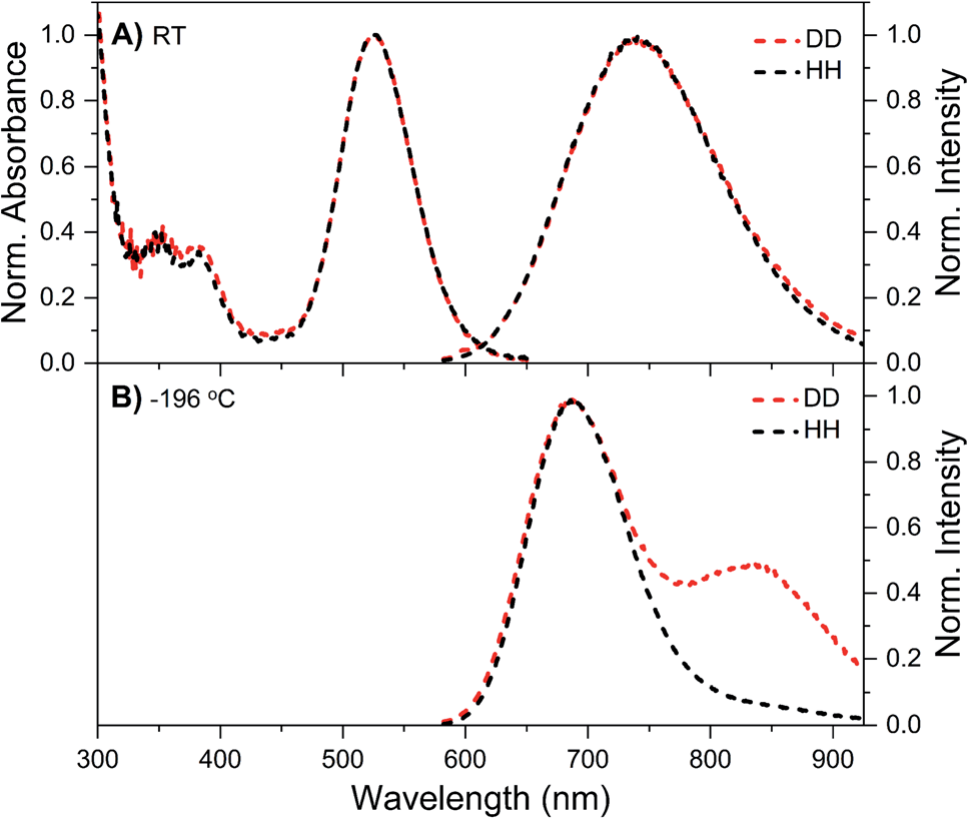
Steady-state spectra of DNA–Ag_16_NCs synthesized and measured in a 10 mM NH_4_OAc H_2_O solution (black, defined as HH condition) or synthesized and measured in a 10 mM NH_4_OAc D_2_O solution (red, referred to as DD condition). (A) Normalized absorption (at the 525 nm peak) and emission spectra measured at room temperature (RT). (B) Normalized emission spectra measured at liquid nitrogen temperature (−196 °C) in a cryogenic Linkam stage. All emission spectra were recorded on a single molecule sensitive confocal microscope,^16^ exciting at 520 nm. Note that the spectra in Fig. 1B contain some minor spectral deformations due to difficulties in recording a proper intensity calibration spectrum in this configuration. See Fig. S2† for details.

Details on the synthesis, HPLC purification and the collected fraction of the DNA–Ag_16_NC sample can be found in the ESI and Fig. S1.†Fig. 1A shows the normalized absorption and emission spectra of DNA–Ag_16_NCs in the DD condition (synthesized and measured in a 10 mM NH_4_OAc D_2_O solution) and HH condition (synthesized and measured in a 10 mM NH_4_OAc H_2_O solution) at room temperature. Note that we did not use deuterated ammonium acetate (ND_4_OAc) because the concentration of H_2_O present as impurity in D_2_O (0.1% of 55 M D_2_O) is in the same order of magnitude as the concentration of NH_4_OAc (10 mM). The absorption features of the main 525 nm peak are identical for the DD and HH conditions, and only a minor offset can be seen at wavelengths below 470 nm. The emission spectra at room temperature look also similar, with a slightly more pronounced red edge in the DD condition.

**Fig. 2:**
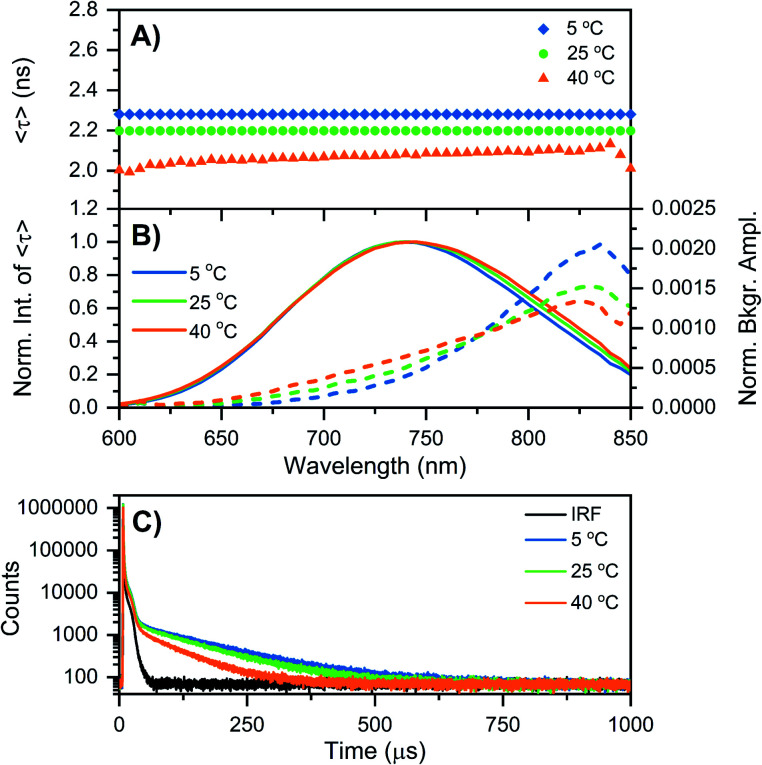
Time-resolved measurements of DNA–Ag_16_NCs synthesized and measured in a 10 mM NH_4_OAc D_2_O solution, performed at three different temperatures: 5, 25, and 40 °C. (A and B) The sample was excited at 531 nm with a ps-pulsed laser. The recorded decay curves were globally fitted with a mono-exponential function at 5 and 25 °C, and a bi-exponential model at 40 °C. (A) Intensity-averaged decay time, 〈*τ*〉, as a function of emission wavelength. (B) Normalized emission intensity of 〈*τ*〉 (solid lines) and background amplitude of the decays (dashed lines) as a function of emission wavelength.^14^ The background amplitudes (a proxy for the µs-lived emission) were normalized by the corresponding emission intensity maxima of 〈*τ*〉. (C) Decay curves recorded at 810 nm, exciting at 531 nm with a Xe flash lamp (repetition rate = 300 Hz). The black curve is the instrument response function (IRF).

**Fig. 3:**
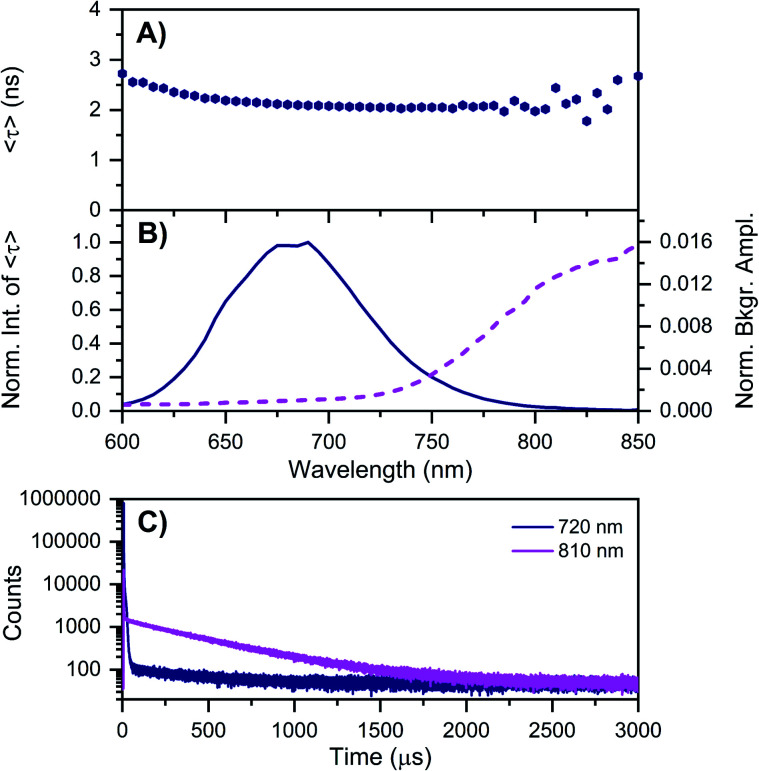
Time-resolved measurements of DNA–Ag_16_NCs synthesized and measured in a 10 mM NH_4_OAc D_2_O solution, carried out at −196 °C. See ESI† for details. (A) 〈*τ*〉 and (B) corresponding emission intensity (solid line) and background amplitude of the decays (dashed line) as a function of emission wavelength.^14^ The background amplitudes (a proxy for the µs-lived emission) were normalized by the corresponding 〈*τ*〉 emission intensity maxima. The sample was excited at 531 nm with a ps-pulsed laser. (C) Microsecond decay curves recorded at 720 nm (dark blue) and 810 nm (magenta), exciting at 531 nm with a Xe flash lamp (repetition rate = 300 Hz).

While at first sight this minor deviation might look unimportant, it manifests itself at −196 °C as an additional emission band centered around 850 nm (Fig. 1B). When performing time-correlated single photon counting (TCSPC) measurements in the DD condition at 5, 25, and 40 °C, the background amplitude in the decay curves increases from 600 nm to 850 nm (dashed traces, Fig. 2B). This indicates the presence of a long-lived and red-shifted luminescence.^14^ The ns-lived emission intensities are reported as solid traces in Fig. 2B. At 5 and 25 °C the decay curves can be fitted satisfactory with a mono-exponential function and only a very small nanosecond (slow) spectral relaxation^12,17,18^ is observed at 40 °C (Fig. 2A). Moreover, the emission intensity decreases by increasing the temperature, as shown in the steady-state spectra reported in Fig. S3 (see also Fig. S9A in ref. 12 for HH condition).† TCSPC data, *e.g.* in Fig. 2B, were recorded with different laser repetition rates and emission attenuations, therefore the intensities of 〈*τ*〉 were normalized and the background amplitudes were rescaled dividing by the corresponding 〈*τ*〉 emission intensity maxima (the same normalization was also performed for Fig. 3, S4 and S5†). The background amplitude seems to reach a maximum around 830 nm, but it should be noted that given the drop in the detector sensitivity above 800 nm, the spectral shape might not be represented correctly. However, it still gives a good indication of where the long-lived emission is spectrally located.

Similar results were found when DNA–Ag_16_NCs were first synthesized in H_2_O and then the solvent was changed to D_2_O for the measurements (see Fig. S4 and Section 3 in the ESI†). Fig. 2C shows decay curves detected at 810 nm in the DD condition, exciting at 531 nm with a Xe flash lamp (repetition rate = 300 Hz). A bi-exponential tail fit was used to determine the µs decay time (〈*τ*_µs_〉) in the 5 to 40 °C range (Table 1). When DNA–Ag_16_NCs are measured in H_2_O, the amount of µs-lived emission at 25 °C can be considered negligible, as shown in Fig. S5B and S6.†

Overview of the time-resolved photophysical properties of DNA–Ag_16_NCs

**Table:** 

DNA–Ag_16_NC (5′-CACCTAGCGA-3′)				
Temp. (°C)	Solvent^a^	〈*τ*〉^b^ (ns) (740 nm)	Solvent^a^	〈*τ*〉^b^ (ns) (740 nm)
−196	HH	5.22^c^	DD	2.12^c^
5	HH	3.73	DD	2.28
	HH_50_/D_50_	2.91	DH_50_/D_50_	2.84
	DH	3.62	HD	2.30
25	HH	3.23	DD	2.20
	HH_50_/D_50_	2.60	DH_50_/D_50_	2.62
	DH	3.16	HD	2.21
40	HH	2.79	DD	2.07
	HH_50_/D_50_	2.39	DH_50_/D_50_	2.37
	DH	2.73	HD	2.08

a HH: synthesized and measured in a 10 mM NH_4_OAc H_2_O solution. DD: synthesized and measured in a 10 mM NH_4_OAc D_2_O solution. HD: synthesized in 10 mM NH_4_OAc H_2_O solution and measured in a 10 mM NH_4_OAc D_2_O solution. DH: synthesized in 10 mM NH_4_OAc D_2_O solution and measured in a 10 mM NH_4_OAc H_2_O solution. HH_50_/D_50_: synthesized in a 10 mM NH_4_OAc H_2_O solution and measured in a 10 mM NH_4_OAc 1 : 1 H_2_O : D_2_O solution. DH_50_/D_50_: synthesized in a 10 mM NH_4_OAc D_2_O solution and measured in a 10 mM NH_4_OAc 1 : 1 H_2_O : D_2_O solution.

b Intensity-weighted average decay times 〈*τ*〉, obtained from decay curves recorded at the indicated emission wavelength (*λ*_exc_ = 531 nm).

c Decays measured at 720 nm, since the emission maximum is blue-shifted at −196 °C.

d Microsecond intensity-weighted average decay times 〈*τ*_µs_〉, obtained from decay curves recorded at the indicated emission wavelength, exciting at 531 nm with a Xe flash lamp (repetition rate = 300 Hz).

e <: amplitude too low to determine the decay time.

f IRF: IRF-limited decay time. Graphical representations of the data can be found in Fig. S9–S11.

**Table:** 

Temp. (°C)	Solvent^a^	〈*τ*_µs_〉^c^^,^^d^ (µs) (720 nm)	〈*τ*_µs_〉^d^ (µs) (810 nm)
−196	HH	<^e^	245
25		IRF^f^	IRF^f^
−196	DD	<^e^	447
5		117	133
25		105	112
40		67	72

As mentioned above, freezing DNA–Ag_16_NCs with liquid nitrogen (−196 °C) makes the µs-lived emission band (*λ*_max_ ≈ 850 nm) much more pronounced. The ns-lived fluorescence is blue-shifted for both DD and HH conditions with a maximum around 690 nm. When performing time-resolved measurements with a Xe flash lamp (repetition rate = 300 Hz) at −196 °C (Fig. 3C, S7† and Table 1), µs-lived emission can be observed for both the DD and HH conditions. The long-lived emission is more pronounced for the DD condition with 〈*τ*_µs_〉 = 447 µs, in line with the steady-state results presented in Fig. 1B. A decay time of 245 µs was instead found for DNA–Ag_16_NCs in the HH condition. In addition, based on a recent article by Petty *et al.*,^15^ we decided to measure the µs-lived emission of DNA–Ag_16_NCs in the DD condition at −196 °C using the burst excitation mode with a picosecond-pulsed laser (IRF ≈ 150 ps).^19,20^

The results can be found in Fig. S8† and very similar values (ranging from 445 to 457 µs for both the rise and decay times of the µs-lived emission) were obtained. So far, the presented data shows that the µs-lived emissive state is more clearly visible in D_2_O with respect to H_2_O. Is this because the long-lived emissive state is less quenched in D_2_O than H_2_O, or that D_2_O promotes the formation of the long-lived state, or a combination of both? Qualitatively, the reduced quenching by D_2_O (〈*τ*_µs_〉 is 1.82 times longer at −196 °C) is not enough to explain the much larger intensity increase of the 850 nm emission in DD *versus* HH condition (Fig. 1B). Hence, D_2_O must increase both the quantum yield of formation of the µs-lived state and its decay time compared to H_2_O. This seems to agree with the finding that the quantum yield of fluorescence is lower in the DD condition *versus* the HH condition (Fig. S9 and Table S2†). The mechanistic origin of why deuterium would enhance the transition to the µs-lived state is currently not understood, but we speculate that differences in the vibrational frequency of the X–D *versus* the X–H bonds (X being nitrogen or oxygen) and/or changes in the strength of the hydrogen bonding network^9,21^ promote the formation of a Frank–Condon (FC) state that increases the likelihood of µs-lived state formation.

The unusual effect of deuterium on DNA–Ag_16_NCs can also be seen in the ns-lived emission. Contrary to the vast majority of fluorophores,^5,8^ 〈*τ*〉 in the DD condition is shorter than that in HH at all temperatures (see Table 1). To confirm that hydrogen and deuterium can dynamically exchange in the DNA–Ag_16_NC structure, two additional conditions were created and measured (HD and DH, see Table 1 for description). Table 1 shows that the solvent used during the synthesis of DNA–Ag_16_NCs is not important for the observed behavior; only the solvent in which the measurements are performed determines the spectroscopic properties. In addition, when DNA–Ag_16_NCs were measured in a 1 : 1 mixture of D_2_O and H_2_O (DH_50_/D_50_ or HH_50_/D_50_), 〈*τ*〉 and *Q* were found to be in between the DD and HH values (Tables 1, S2 and Fig. S9–S11†). While overall smaller, the ns decay time in D_2_O was significantly less temperature-dependent than in H_2_O (Table 1).

Based on available data from organic fluorophores,^5^ it is rather unlikely that the shortening of the ns-lived state can be attributed to a more efficient non-radiative quenching by D_2_O *versus* H_2_O. Instead, the origin of the lower ns-decay time in D_2_O must be related to another effect. We have observed previously for a red-emissive DNA–AgNC that, when immobilized in a polymer film and studied at the single molecule level, 〈*τ*〉 is oppositely correlated with the ability to form long-lived (µs) dark states.^22^ In the latter case, the dark state formation was probed by optically activated delayed fluorescence (OADF).^22^ Intriguingly, Fig. 3A shows that 〈*τ*〉 at −196 °C increases from 650 nm (2.19 ns) to 600 nm (2.72 ns). This might indicate the presence of a blue-shifted population that has a longer decay time and perhaps less µs-lived state formation. Future excitation-wavelength dependent ratiometric studies of the ns-lived *versus* µs-lived emission could potentially shed light on this. When DNA–Ag_16_NC are frozen in the HH condition, 〈*τ*〉 is significantly longer (5.22 ns), as is usually observed when suppressing temperature-dependent non-radiative decay pathways.

### DNA721–AgNC

**Fig. 4:**
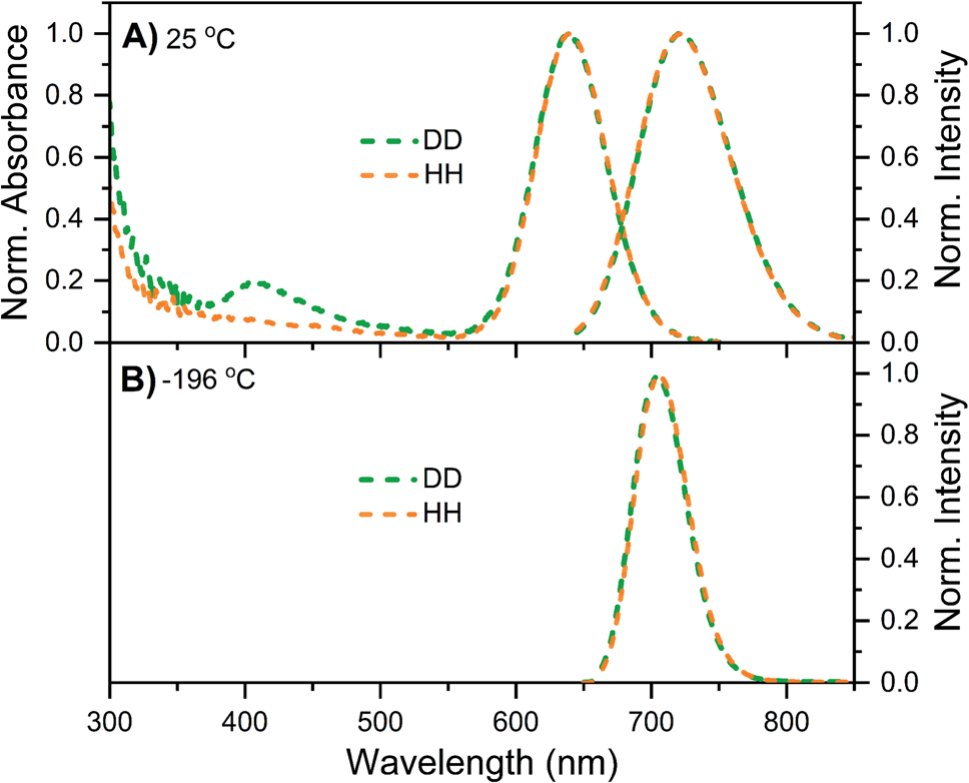
Steady-state data of DNA721–AgNCs in DD (green) and HH (orange) conditions. (A) Normalized absorption (at the 640 nm peak) and emission spectra at 25 °C. (B) Normalized emission spectra recorded in liquid nitrogen (−196 °C). All emission spectra were recorded with a Fluotime300 instrument, exciting at 634.8 nm with a ps-pulsed laser.

DNA721–AgNC emits in the same range as DNA–Ag_16_NC, but has a distinctly different spectroscopic behavior.^13^ Details on the synthesis, HPLC purification and the collected DNA721–AgNC fraction can be found in the ESI and Fig. S13.†Fig. 4A shows the normalized absorption and steady-state emission spectra of DNA721–AgNCs in the DD and HH conditions. Both emission spectra, as well as the 640 nm absorption features, are identical and overlap perfectly. The only difference is an extra absorption band around 400 nm in the DD condition, which was not previously observed in H_2_O.^13^ This feature is absent in the excitation spectrum (Fig. S14†), thus we can exclude that D_2_O induces an additional electronic transition at 400 nm. It is instead very likely that the absorption bump for the DD condition is due to the presence of an impurity collected during the HPLC run. Freezing the DNA721–AgNC sample with liquid nitrogen in the DD and HH conditions blue-shifts the emission maximum to 706 nm (Fig. 4B).

TCSPC measurements yielded differences in 〈*τ*〉 (see Table 2) for the DD *versus* HH condition. For example, at 25 °C, 〈*τ*〉 is 4.36 ns in the DD condition and 3.72 ns for the HH condition. Unlike DNA–Ag_16_NCs, D_2_O seems to affect the behavior of DNA721–AgNCs in a more expected way, lengthening 〈*τ*〉 and increasing *Q*.^5^ For both the HH^13^ and DD conditions, 〈*τ*〉 is rather temperature-independent in the 5–40 °C range, while the emission intensity and absorbance are temperature-dependent. This means that 〈*τ*〉 and *Q* are not interdependent and the classic three-level model where *Q* = *k*_f_〈*τ*〉 (*k*_f_ being the radiative rate constant) does not apply unless one introduces static quenching.^23^ We have previously suggested that for some DNA–AgNCs, a phenomenological four-level model, originally introduced by Patel *et al.*,^24^ can explain the observed relationship between *Q* and 〈*τ*〉.^17,25^ In this model, the DNA–AgNC is excited into a FC state that evolves ultrafast (sub-ps)^24^ either back to the ground state, a µs-lived state, or the ns-lived emissive state. The first two pathways (to the ground state and to the µs-lived state) can be considered as a type of static quenching with regard to the emission from the ns-lived state.

Overview of the steady-state and time-resolved photophysical properties of DNA721–AgNCs

**Table:** 

DNA721–AgNC (5′-CCCGGAGAAG-3′)				
Temp. (°C)	Solvent^a^	〈*τ*〉^b^ (ns) (720 nm)	Solvent^a^	〈*τ*〉^b^ (ns) (720 nm)
−196	HH	3.64	DD	3.79
5	HH	3.75^c^	DD	4.39
	DH	3.81	HD	4.29
25	HH	3.72^c^	DD	4.42
	HH_50_/D_50_	4.01	DH_50_/D_50_	4.06
	DH	3.77	HD	4.33
40	HH	—e	DD	4.47
	DH	3.73	HD	4.37

a See Table 1 caption for the explanation of HH, HD, DH, DD, HH_50_/D_50_ and DH_50_/D_50_ abbreviations.

b Intensity-averaged decay times 〈*τ*〉, obtained from decay curves recorded at the indicated emission wavelength (*λ*_exc_ = 634.8 nm).

c Data taken from ref. 9.

d *Q* value used as reference for the determination of the quantum yield of DNA721–AgNCs in different solvent and temperature conditions (see Fig. 5, ESI and Fig. S15 for more details). Note: changes in the refractive index (both temperature and isotope changes) were ignored since they are in the 1% difference range. A graphical representation of 〈*τ*〉 data as a function of temperature can be found in Fig. S16.

e — data not measured.

**Table:** 

Temp. (°C)	Solvent^a^	*Q*	Solvent^a^	*Q*
5	HH	—e	DD	1.00
	DH	0.82	HD	0.94
25	HH	0.73^c^^,^^d^	DD	0.91
	HH_50_/D_50_	0.78	DH_50_/D_50_	0.81
	DH	0.72	HD	0.86
40	HH	—e	DD	0.86
	DH	0.68	HD	0.86

Hence, *Q* becomes the product of *Q*_S1_ (quantum yield of ns-lived state formation) and *Q*_f_ (the quantum yield of fluorescence from the emissive state to the ground state).^17^ Since 〈*τ*〉 is largely temperature-independent, the main cause for changes in *Q* is the temperature-dependent change of *Q*_S1_.

We tried to test this hypothesis by plotting 〈*τ*〉 as a function of *Q* calculated for different solvent and temperature conditions. *Q* values were determined using the previously reported 0.73 (HH condition at 25 °C) as reference value.^13^Fig. 5 shows that 〈*τ*〉 *vs. Q*, for both H_2_O and D_2_O measurement conditions, follows a linear trend that does not intercept the origin (0,0).

**Fig. 5:**
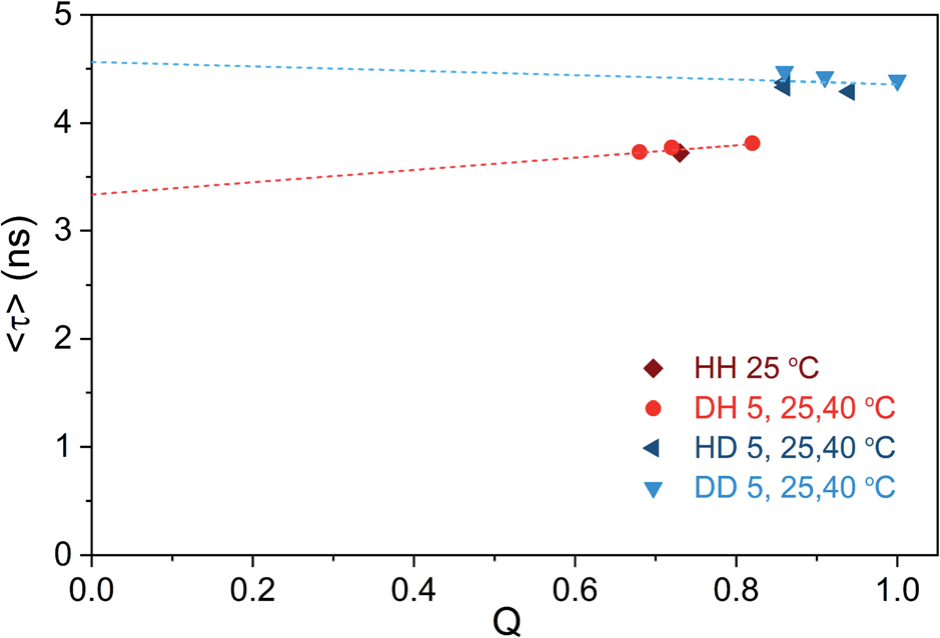
Intensity-weighted average decay time 〈*τ*〉 of DNA721–AgNCs as a function of fluorescence quantum yield (*Q*), for different solvent and temperature conditions. The HH condition at 25 °C was used as the reference quantum yield (0.73),^13^ and the other *Q* values were determined from single emission and absorption spectra at the specified condition (Fig. S15†).^26^ Note that changes in the refractive index (both temperature- and isotope-dependent changes) were ignored since they are in the 1% difference range. The dashed lines represent the linear fit of the blue and red data points, respectively.

It is also worth noticing that *Q* for the DD condition at 5 °C reaches unity. While Fig. 5 illustrates that the “classic” three-level model is not applicable for DNA721–AgNCs, Petty *et al.* have recently demonstrated that the dual emission of a green- and NIR-emitting DNA–AgNC can be described by this model.^15^ The latter highlights the need for electronic structure calculations in order to help interpret the experimental data from different DNA–AgNCs.^2^

Unlike DNA–Ag_16_NC, DNA721–AgNC displays no significant µs-lived emission either in the liquid (*e.g.* 5 to 40 °C) or the frozen (−196 °C) state. This is in line with the steady-state data in Fig. 4 where no additional emission band up to 850 nm appears. However, we have previously reported^13^ that DNA721–AgNCs can form long-lived states that can be optically depleted yielding OADF.^13,25,27^ This means that the µs-lived states in DNA721–AgNC are either dark or emit in a NIR range significantly beyond our detection window. While no crystal structure information is available for DNA721–AgNC, its hydrodynamic volume (19.6 nm^3^)^13^ is significantly larger than that of DNA–Ag_16_NC (10.5 nm^3^).^12^ The two DNA–AgNCs could have different levels of solvent accessibility to the AgNC, but in both cases the final measurement solvent determines the properties, indicating reasonable exchangeability for both.

## Conclusions

We have shown that exchanging H_2_O with D_2_O as solvent can have diverse effects for different DNA–AgNCs. For DNA–Ag_16_NCs, 〈*τ*〉 shortened, which is in contrast with the behavior observed for DNA721–AgNCs and most organic fluorophores.^5^ D_2_O also enhanced the formation of the µs-lived state and lengthened 〈*τ*_µs_〉 for DNA–Ag_16_NCs. While the mechanistic origin is not understood, we hypothesize that the difference in the vibrational frequencies of the X–D *versus* the X–H bonds and/or changes in the strength of the hydrogen bonding network affect the excited state pathways. For DNA721–AgNCs, D_2_O lengthened 〈*τ*〉 and increased *Q* compared to H_2_O. At 5 °C in D_2_O, *Q* even approaches unity.

Furthermore, we have demonstrated for DNA721–AgNCs that the temperature-dependent changes of *Q* are not reflected in the 〈*τ*〉 values. This indicates that the variations in *Q* are not due to the changes in the non-radiative decay rates from the emissive state, but are mostly caused by the changes in the quantum yield of the emissive state formation (*Q*_S1_).^17^ We hope that our results will stimulate further research in obtaining high fluorescence quantum yield NIR-emitting DNA–AgNCs.

## Data availability

Experimental data and details on the experimental procedures are provided in the ESI.†

## Author contributions

C. C. synthesized and purified the DNA-AgNCs. C. C. and G. P.-H. performed most of the steady-state and time-resolved experiments. M. B. L. recorded the emission spectra on the microscope. T. V. and C. C. conceived the experiments. The paper was written with contributions from C. C., G. P.-H., M. B. L. and T. V.

## Conflicts of interest

There are no conflicts to declare.

## Note added after first publication

This version replaces the manuscript published on 25th November 2021 which contained an incomplete caption for Figure 2. The correct and complete caption is now provided above. The RSC apologises for any confusion.

## Supplementary Material

SC-012-D1SC05079F-s001
