# Supplementary material for: The effect of deuterium on the photophysical properties of DNA-stabilized silver nanoclusters
Source: Chem Sci. 2021 Nov 25;12(48):16100–5. doi: 10.1039/d1sc05079f (PMC8672707; doi:10.1039/d1sc05079f)
Supplement: SC-012-D1SC05079F-s001 [file SC-012-D1SC05079F-s001.pdf]

Electronic Supplementary Material (ESI) for Chemical Science.
This journal is © The Royal Society of Chemistry 2021

Supporting Information.

The effect of deuterium on the photophysical properties of DNA-stabilized silver nanoclusters.

Cecilia Cerretani,* Gustav Palm-Henriksen, Mikkel B. Liisberg, and Tom Vosch*

Department of Chemistry, University of Copenhagen, Universitetsparken 5, 2100 Copenhagen, Denmark. Email: cece@chem.ku.dk; tom@chem.ku.dk

MATERIALS AND METHODS

1. Synthesis of DNA-Ag_{16}NC and DNA721-AgNC

DNA-Ag_{16}NCs and DNA721-AgNCs were synthesized in two different media:

i. 10 mM ammonium acetate H_2O solution (defined as H)

ii. 10 mM ammonium acetate D_2O solution (defined as D)

For the synthesis performed in H, the DNA was dissolved in nuclease-free H_2O and all solutions were prepared in the same medium. For the synthesis carried out in D, the DNA was dissolved in D_2O and all solutions were prepared in the same solvent.

DNA oligomers and nuclease-free H_2O were purchased from Integrated DNA Technologies, D_2O (99.90%) was purchased from Eurisotop, while $AgNO_3$ (≥ 99.998%), ammonium acetate (NH_4OAc, ≥ 98%) and $NaBH_4$ (≥ 99.99%) were acquired from Sigma Aldrich.

DNA-Ag_{16}NCs (5'-CACCTAGCGA-3') and DNA721-AgNCs (5'-CCCGGAGAAG-3') were prepared by mixing the DNA oligonucleotides with $AgNO_3$ in 10 mM ammonium acetate at pH 7. After 15 minutes $NaBH_4$ was added in order to reduce the silver cations and promote the formation of silver clusters.

For DNA-Ag_{16}NCs, the ratio between the components was [DNA]: [$AgNO_3$]: [$NaBH_4$] = 25 μM: 187.5 μM: 93.75 μM, whereas for DNA721-AgNCs, the chosen DNA concentration was 20 μM, and the final ratio was 1: 5: 2.5. After synthesis, the solutions were stored in the fridge. For DNA-Ag_{16}NCs, the HPLC purification was performed 3 days after the synthesis, while DNA721-AgNCs were purified after 5 days.

In the end, the purified fractions were upconcentrated and solvent-exchanged to a 10 mM NH_4OAc H_2O or D_2O solution by spin-filtration (3 kDa cut-off membrane filters), based on the solvent used in the synthesis.

2. HPLC purification

For both DNA-AgNCs, the HPLC purification was performed using a preparative HPLC system from Agilent Technologies with an Agilent Technologies 1260 Infinity fluorescence detector, an Agilent Technologies 1100 Series UV-Vis detector, and a Kinetex C18 column (5 μm, 100 Å, 250 × 4.6 mm), equipped with a fraction collector. The mobile phase was a gradient mixture of 35 mM triethylammonium acetate (TEAA) buffer in water (A) and methanol (B). The flow rate was 1 mL/min.

For DNA-Ag_{16}NCs synthesized in 10 mM NH_4OAc H_2O and D_2O solutions, the elution gradient ranged from 15% to 95% B in 24 min. In the first 2 min, it was varied from 15 to 20% B, then it was linearly increased to 40% B in 20 min, and finally in the 22-24 min interval, the gradient was rapidly risen to 95% B. The collection was based on the absorbance at 530 nm. The run was followed by 6 min of washing with 95% B to remove any remaining sample from the column. As shown in the chromatograms in Figure S1, the purified fractions eluted around 16.5-18 min (≈34.5-36% B).

For DNA721-AgNCs, slightly different methods were used for both H and D conditions compared to that described in reference 4. Details are reported in Table S1.

DNA721-AgNCs in 10 mM NH_4OAc **H_2O** solution		DNA721-AgNCs in 10 mM NH_4OAc **D_2O** solution	
Time (min)	35 mM TEAA in MeOH (B)	Time (min)	35 mM TEAA in MeOH (B)
0-2	10%	0-2	10%
2-22	10-30%	2-27	10-35%
22-25	30-95%	27-30	35-95%

Table S1. HPLC purification methods/elution gradients for DNA721-AgNCs synthesized in 10 mM NH_4OAc H_2O and D_2O solutions.

Every run was followed by 5 min of washing with 95% B. Fraction collection was carried out using the absorbance signal at 640 nm. Figure S13 shows the HPLC chromatograms for both solutions. The purified fraction synthesized in H was collected between 14 and 16 min (*i.e.* 22-24% B), while the DNA721-AgNCs prepared in D eluted in the 14.6-20 min interval (≈22.6-28% B). The broad and unstructured peak could be the reason for the 400 nm absorption band in the D condition (Figure 4A). It is likely that some impurities/side products were collected along with the main NIR emissive species.

3. Spectroscopic measurements

Steady-state and time-resolved measurements for DNA-Ag_{16}NCs and DNA721-AgNCs were carried out in different conditions: HH, HH_{50}/D_{50}, HD, DH, DH_{50}/D_{50} and DD, and diverse temperatures: -196, 5, 25, and 40 ºC. Measurements at -196 ºC will be described in paragraph 3.2.5. The first H or D represents the solvent where the DNA-AgNCs were synthesized in (see section 1), whereas H, H_{50}/D_{50}, and D placed afterwards define the medium where the measurements were performed. H_{50}/D_{50} is a 10 mM NH_4OAc 1:1 H_2O:D_2O solution. HD, DH, HH_{50}/D_{50}, DH_{50}/D_{50} conditions were prepared by dissolving a very tiny volume of concentrated DNA-AgNC stock solution (~10-20 µL) into the final solvent (~2 mL). Preliminary experiments showed that this gave similar results as a solvent exchange using spin-filtration.

3.1 Absorption measurements

Absorption spectra were measured with a Cary 300 UV-Vis spectrophotometer from Agilent Technologies using a deuterium lamp for ultraviolet radiation and a tungsten-halogen lamp for visible and near-infrared (NIR) radiation.

3.2 FluoTime300 instrument (PicoQuant)

3.2.1 Steady-state emission measurements

Steady-state fluorescence spectra of DNA-Ag_{16}NCs and DNA721-AgNCs were recorded exciting the samples with picosecond-pulsed lasers (PicoQuant): 531 nm (LDH-D-TA-530B) and

634.8 nm (LDH-P-635), respectively. All emission spectra have been corrected for the wavelength dependency of the detector.

3.2.2 Quantum Yield (Q) measurements and calculations

Quantum yield values for DNA-Ag_{16}NCs and DNA721-AgNCs were determined in different solvent and temperature conditions by a relative method,[1] using the value in the HH condition at 25 ºC as reference (Q=0.26 for DNA-Ag_{16}NCs,[2] and 0.73 for DNA721-AgNCs[3]). Only one point for the integrated emission counts and one point for the fraction of absorbed light were utilized in the calculations (See Figure S15 for the actual data of DNA721-AgNCs).

$$Q_{NC} = \frac{F_{NC}}{f_{A,NC}} \cdot \frac{f_{A,ref}}{F_{ref}} \cdot \frac{n_{NC}^2}{n_{ref}^2} \cdot Q_{ref} \quad \textbf{(1)}$$

where:

- **Q** represents the quantum yield;
- **F** is the integrated emission spectrum (*i.e.* the area under the fluorescence spectrum);
- $\mathbf{f_A}$ is the fraction of absorbed light at the excitation wavelength (λ_{exc}=531 nm for DNA-Ag_{16}NCs, and 635 nm for DNA721-AgNCs);
- **n** is the refractive index of the medium where the clusters were dissolved in during the measurements. Changes in the refractive index due to temperature and isotope variations are ignored, since they are in the 1% difference range.

The subscripts **NC** indicates the DNA-AgNCs in the solvent and temperature of interest, while **ref** represents the corresponding DNA-AgNCs in HH condition at 25 ºC.

3.2.3 Excitation spectra measurements

Excitation spectra were measured for DNA721-AgNCs with a Xe lamp, monitoring the emission at 720 nm. The spectra were recorded in DD and HH conditions at 25 ºC, and then corrected for the wavelength dependency of the detector and for the lamp power (Figure S14).

3.2.4 Time-correlated single photon counting (TCSPC)

Time-resolved fluorescence measurements for DNA-Ag_{16}NCs and DNA721-AgNCs were performed exciting with a vertically-polarized 531 nm or 634.8 nm pulsed laser, respectively.
Single fluorescence decays were acquired at 740 nm for DNA-Ag_{16}NCs and 720 nm for DNA721-AgNCs. The integration time was chosen between 10 and 90 s in order to reach at least 10,000 counts in the maximum.
The analysis of time-resolved data was performed with FluoFit v.4.6 software from PicoQuant. All decays were fitted with a mono- or multi-exponential reconvolution model including the IRF (instrument response function). The intensity-averaged decay time $\langle\tau\rangle$ was calculated as the intensity-weighted average decay time at the selected emission wavelength.[4]
For DNA-Ag_{16}NCs in DD, HD and DH conditions, decay curves were also measured every 5 nm in the 600-850 nm range at diverse temperatures (5, 25, and 40 ºC). The integration time was

varied between 10 and 20 s, the laser repetition rate was selected from 16.0 to 21.3 MHz, while the emission attenuation was changed between 54% and 68%. These settings were optimized at every temperature in order to reach a trade-off between measurement time and number of counts in the maximum at λ_{em} (at least 10,000). Therefore, the intensity of <τ> has been normalized (divided by the maximum value), since it does not reflect the actual steady-state emission intensity reported in Figure S3.

All decay curves were globally fitted with a multi-exponential reconvolution model including the IRF. The obtained amplitude (α_i) and decay time (τ_i) components were used to calculate <τ> and the corresponding intensity as a function of emission wavelength. Furthermore, the background amplitude of the decays was plotted as a proxy for the microsecond-lived emission, after normalizing the curves for the respective intensity maximum of <τ>. Both the background amplitude and the intensity of <τ> were corrected for the detector efficiency. Figures 2B, 3B, S4B and S5B were constructed based on the following formula:

$$I(\lambda) = Bkgr(\lambda) + \sum_{\lambda}\left(\sum_{i}\alpha_i\tau_i\right)_{\lambda} \quad \textbf{(2)}$$

3.2.5 Steady-state and time-resolved emission measurements at -196 ºC

Low temperature steady-state and time-resolved emission measurements of DNA-Ag_{16}NCs and DNA721-AgNCs were carried out by immersing an NMR tube with the sample in a transparent Dewar filled with liquid nitrogen (-196 °C). The Dewar was then placed in the cuvette compartment of a FluoTime300 instrument from PicoQuant. In order to limit the increased scattering, a band-pass filter in the excitation path and a long-pass filter in the emission path were used. For DNA-Ag_{16}NCs, the two filters were a 527 nm band-pass filter (Semrock, FF01-527/20-25) and a 532 nm long-pass filter (Semrock, BLP01-532R-25). For DNA721-AgNCs, a 640 nm band-pass filter (Semrock, LD01-640/8-25) and a 647 nm long-pass filter (Semrock, BLP01-647R-25) were employed.

The acquired steady-state emission spectra were corrected for the wavelength dependency of the detector.

Low temperature time-resolved data were recorded only for DNA-Ag_{16}NCs and DNA721-AgNCs in HH and DD conditions. Decay curves of DNA-Ag_{16}NCs were measured every 5 nm in the 600-850 nm range. All decay curves were globally fitted with a bi- (HH) or tri-exponential (DD) reconvolution model including the scattered light contribution and the IRF. The obtained amplitude (α_i) and decay time (τ_i) components were used to calculate <τ>, reported in Figure 3A as a function of emission wavelength, while Figure 3B was constructed based on eq. 2. HH data is not shown. For DNA721-AgNCs, fluorescence decays were measured at 720 nm with a 5 s integration time to reach 10,000 counts in the maximum. Each decay was fitted with a bi-exponential reconvolution function including the scattered light contribution and the IRF. The obtained <τ> are reported in Table 2.

3.2.6 Microsecond decay time measurements of DNA-Ag_{16}NC

A) Xe flash lamp

Microsecond decay measurements were performed only for DNA-Ag_{16}NCs in HH and DD conditions at diverse temperatures. The sample was excited with a Xe flash lamp (repetition rate = 300 Hz) at 531 nm. Two-filters were used: a 527 nm band-pass filter (Semrock, FF01-527/20-25) in the excitation path, and a 532 nm long-pass filter (Semrock, BLP01-532R-25) in the emission path.
Decay curves were recorded at 720 nm (-196 and 25 ºC) and 810 nm (5, 25, 40 and -196 ºC), with an integration time of 10 min. All decays and the IRF of the Xe flash lamp are shown in Figures 2C, 3C, S6 and S7. The decays recorded at 720 nm in liquid N_2 for both HH and DD conditions have too low amplitudes to determine the decay time, while both decays measured at 25 ºC in the HH solution are IRF-limited, indicating that no significant microsecond-lived emission is present (Table 1). Every decay measured at 810 nm and the decays at 720 nm in the DD condition at 5, 25, and 40 ºC were tail-fitted bi-exponentially in order to determine the microsecond decay time. The concentration of dissolved molecular oxygen was not actively controlled and the decay time can vary depending on the O_2 concentration (see Figure S12).[5]

B) Burst mode

Emission intensity of DNA-Ag_{16}NCs in the DD condition was measured at 820 nm at -196 °C in burst mode, exciting at 507.5 nm (LDH-P-C-510, PicoQuant). The repetition rate of the laser was set to 40 MHz, with an effective sync rate of 10 Hz. The laser burst lasted 5 ms and consisted of 200,000 pulses (*i.e.* every pulse was separated by 25 ns) followed by 95 ms where the laser was switched off (5% duty cycle). The laser power impinging on the sample was varied from 14 to 86 µW. The integration time was chosen to be 5 min. The intensity build-up of the long-lived emission and the intensity decay upon switching off the laser (at 5 ms) were fitted with a single exponential function. Intensity traces and time constants can be found in Figure S8.

3.3 Home-Built Microscope Setups

The detector of the FluoTime300 system (PicoQuant), used for most measurements, becomes increasingly insensitive beyond 800 nm. To circumvent the low efficiency in the NIR range and to obtain emission spectra with more accurate spectral profiles, we used our home-built confocal microscope for measuring representative emission spectra of DNA-Ag_{16}NCs in HH and DD conditions at room temperature (RT) and -196 °C (Figure 1).[6]
A fiber coupled (NKT Photonics, FD7-PM) continuum white-light laser (NKT Photonics, SuperK EXTREME EXB-6) was used as an excitation source delivering 520 nm wavelength by sending the continuum output through an acousto-optic tunable filter (NKT Photonics, SuperK SELECT). The output of the fiber was cleaned up by a 520 nm band-pass filter (Semrock, FF01-520/5-25) before it was reflected by a 30:70 beam splitter (Omega Optical, XF122) and sent through an objective. For room temperature (RT) measurements, an oil immersion objective (Olympus, UPlanSApo 100x, NA = 1.4) was used to focus the laser onto the sample (20 µL droplet) and to collect the luminescence. For low temperature (-196 °C) measurements, an air objective (Olympus, CPlanFLN 10x, NA = 0.3) was instead used to focus the laser onto the sample (10 µL) positioned on a temperature-controlled stage (LTS 350 and LNP, Linkam) and to collect the luminescence. In both cases, the laser light was blocked by a 561 nm long-pass filter (Semrock, BLP01-561R-25) and out-of-focus light was blocked by a 100 µm pinhole. The luminescence was sent through a spectrograph (Acton Research, SP 2356 spectrometer, 300 grooves/mm) onto a

nitrogen cooled CCD camera (Princeton Instruments, SPEC-10:100B/LN-eXcelon) to record the spectra. As previously reported, the emission spectra were wavelength- and intensity-corrected using a calibrated light source.[6]

DNA-Ag_{16}NC

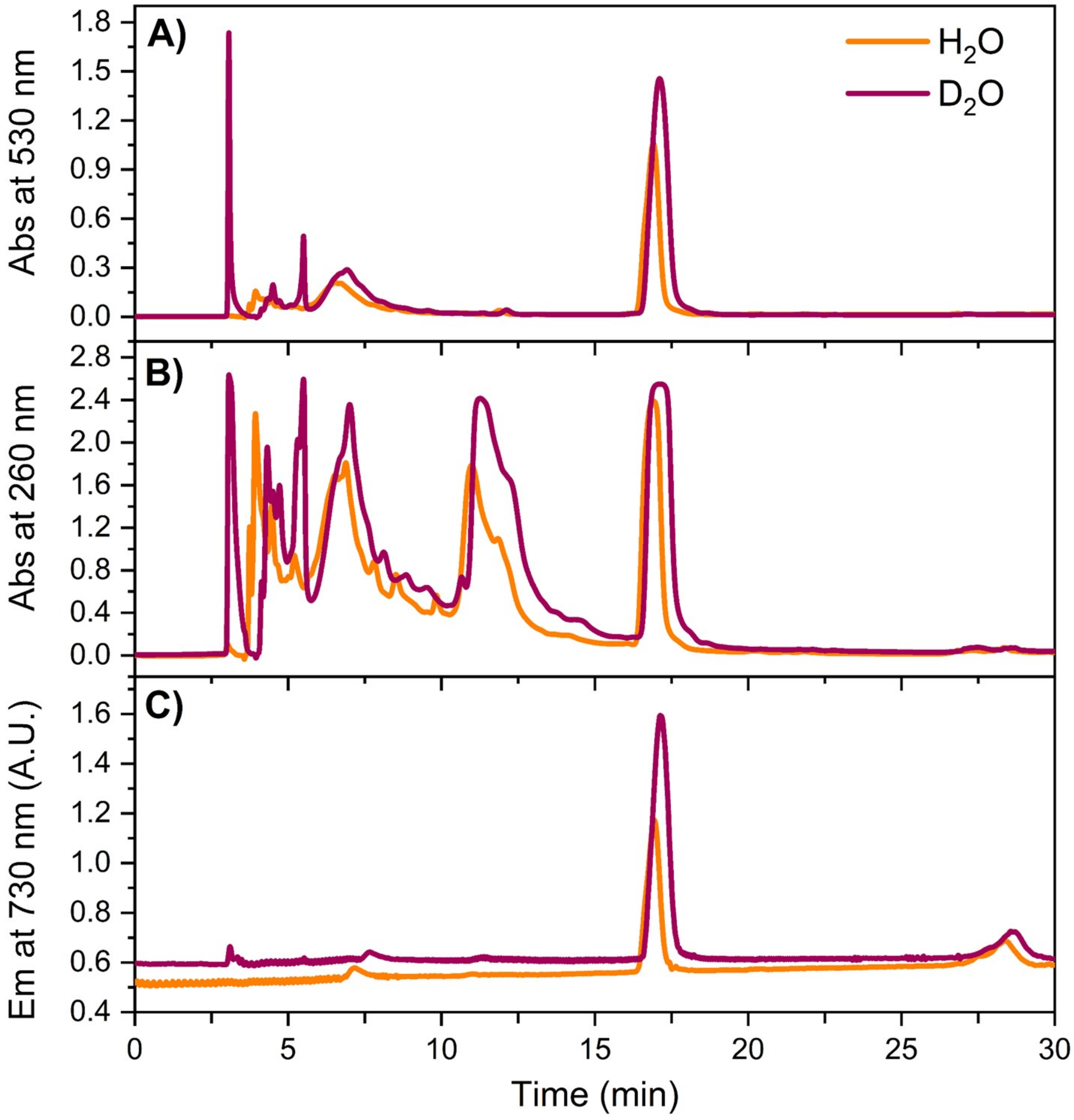

Figure S1. HPLC chromatograms of DNA-Ag_{16}NCs synthesized in 10 mM NH_4OAc H_2O (orange) and D_2O (mauve) solutions **A)** monitoring the main absorption peak of the AgNCs at 530 nm, and **B)** monitoring the DNA absorption at 260 nm. **C)** Chromatograms that monitor the emission of the DNA-Ag_{16}NCs at 730 nm (λ_{exc}= 530 nm). The fractions collected between 16.5-18 min are the samples described in the manuscript.

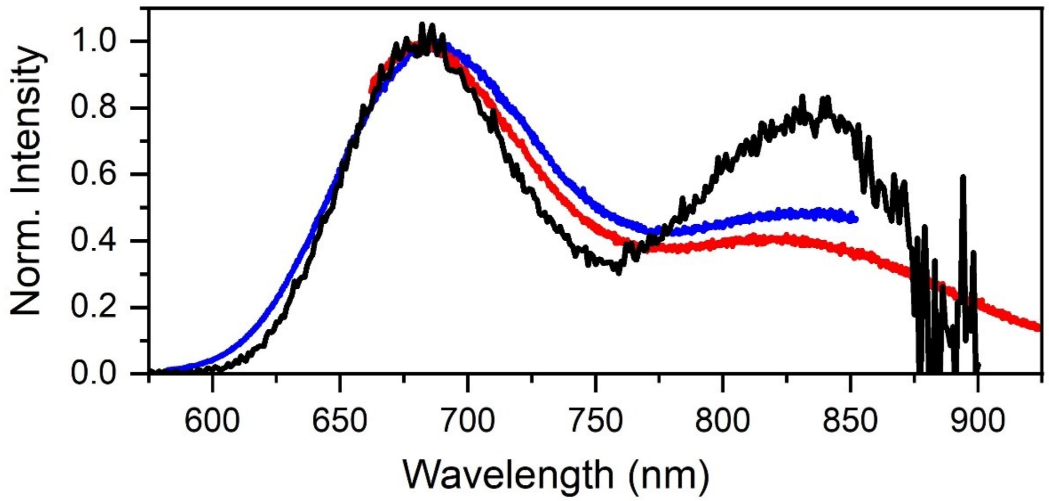

Figure S2. Spectra of DNA-Ag_{16}NCs in the DD condition at -196 °C. The blue are red curves were recorded with a single molecule sensitive microscope[6] with the sample frozen inside a Linkam stage (subsection 3.3). The spectrometer was centered at 720 nm and 800 nm to measure the blue and red spectrum, respectively. Due to difficulties in obtaining a correct calibration spectrum of the calibration lamp in this measurement geometry, both curves do not perfectly overlap as they should. The spectrum shown in Figure 1B is the blue curve until 852 nm, then the red curve was scaled to the same value and the rest of the red curve was used to complete the spectrum. The black curve was acquired with a FluoTime300 instrument, exciting at 531 nm. The sensitivity of the detector drops significantly above 800 nm and despite correcting for this, the spectral shape becomes unreliable in this region. The measurements clearly agree on the 690 nm peak and a second additional band centered around 850 nm. This figure is intended to illustrate the uncertainties on the spectral shape of the sample at -196 °C. (For the solution condition in Figure 1A, there were no problems in obtaining the calibration spectrum of the calibration lamp on the single molecule sensitive microscope.)

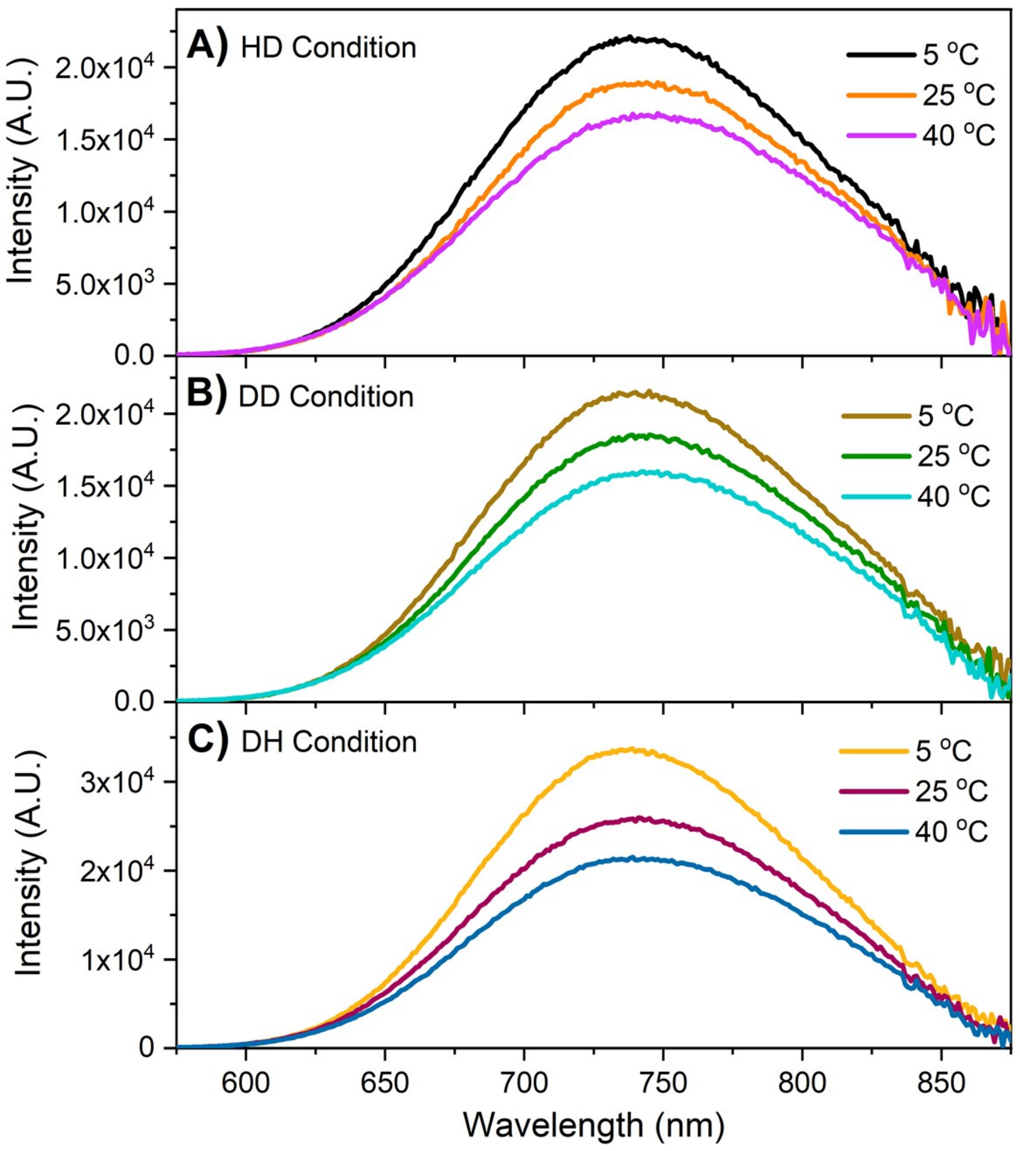

Figure S3. Emission spectra of DNA-Ag_{16}NCs at different temperatures in **A)** HD, **B)** DD, and **C)** DH conditions recorded with a FluoTime300 instrument (λ_{exc}=531 nm).

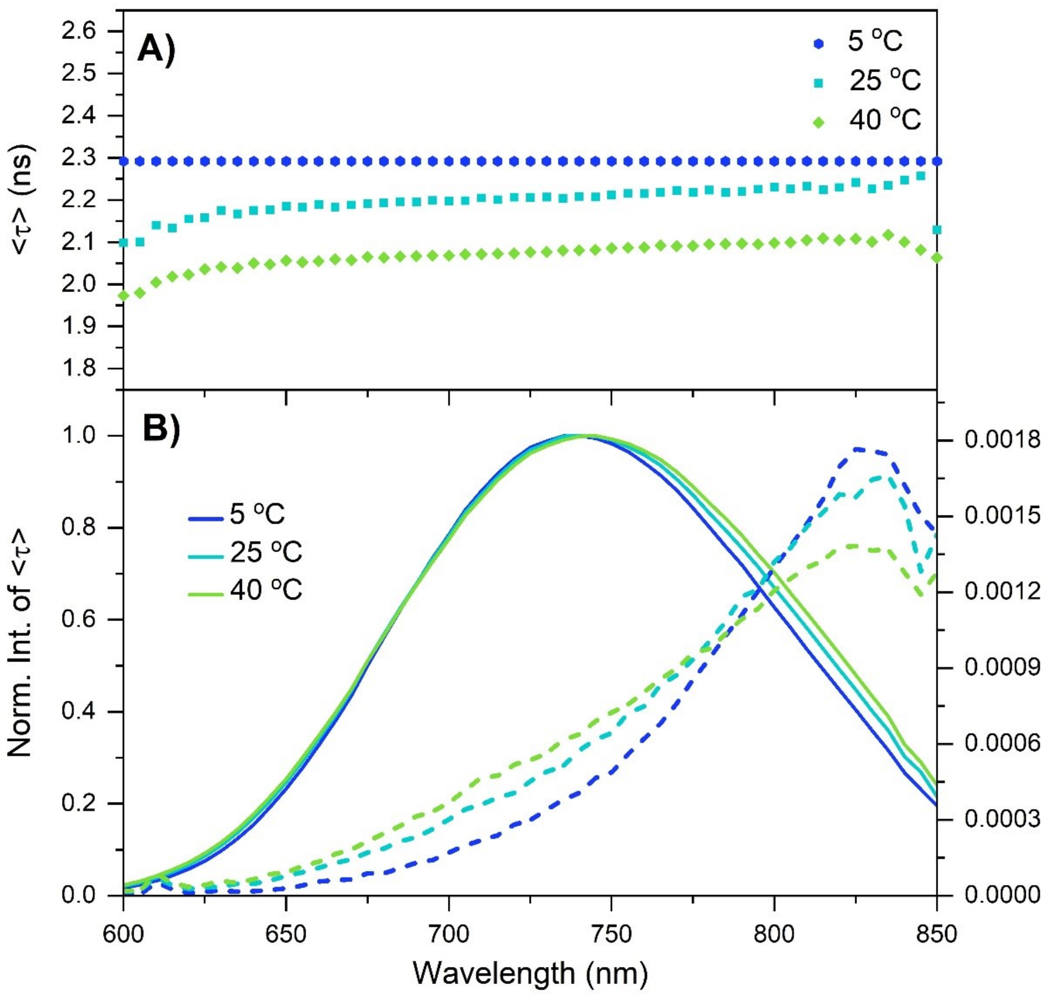

<u>Figure S4.</u> DNA-Ag_{16}NCs in HD condition (synthesized in a 10 mM NH_4OAc H_2O solution and measured in a 10 mM NH_4OAc D_2O solution) at different temperatures. The sample was excited at 531 nm. **A)** Intensity-weighted average decay time (<τ>) as a function of emission wavelength at 5, 25, and 40 ºC. **B)** Normalized emission intensity of <τ> (solid lines) and rescaled background amplitude of the decays (dashed lines) as a function of emission wavelength (see paragraph 3.2.4 for details).

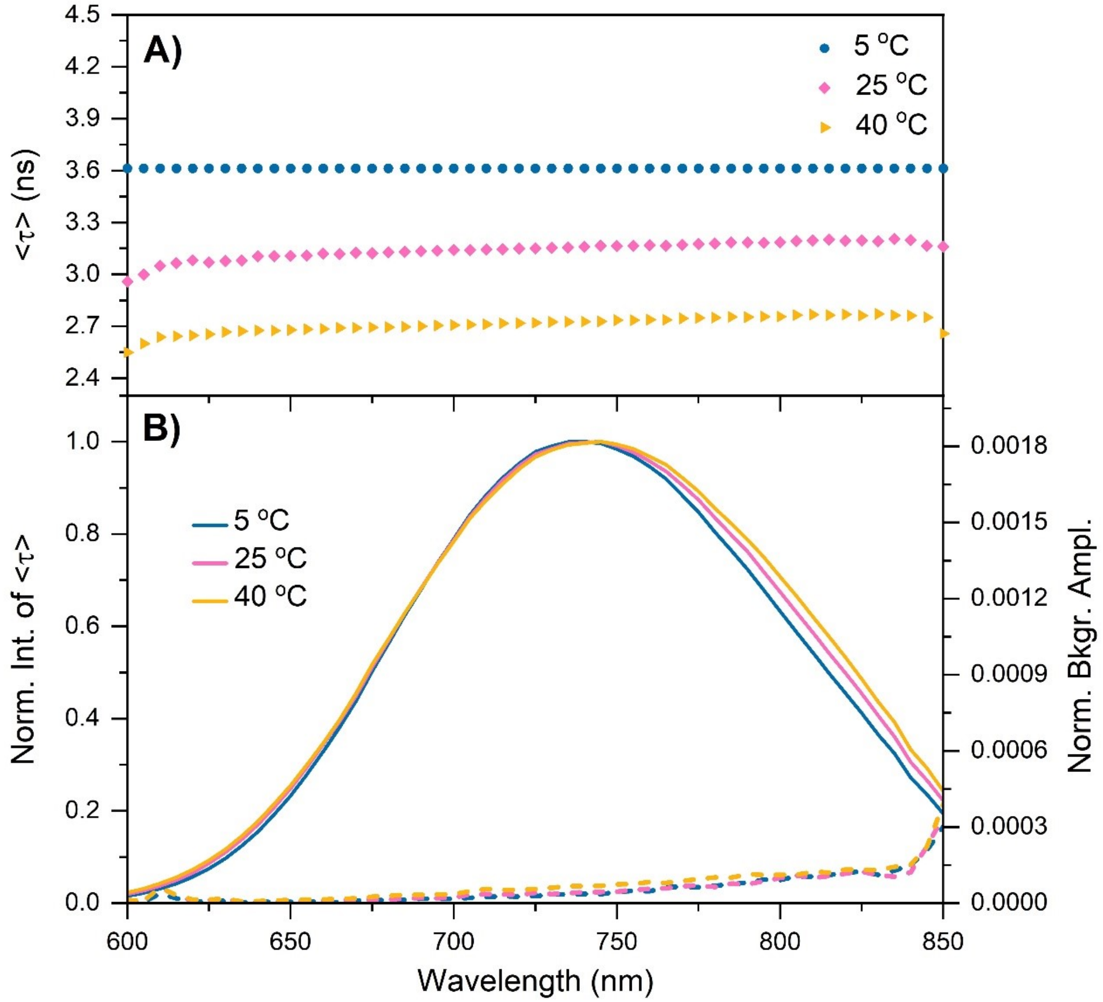

Figure S5. DNA-Ag_{16}NCs in DH condition (synthesized in a 10 mM NH_4OAc D_2O solution and measured in a 10 mM NH_4OAc H_2O solution) at different temperatures. The sample was excited at 531 nm. **A)** Intensity-weighted average decay time (<τ>) as a function of emission wavelength at 5, 25, and 40 ºC. **B)** Normalized emission intensity of <τ> (solid lines) and rescaled background amplitude of the decays (dashed lines) as a function of emission wavelength (see paragraph 3.2.4 for details).

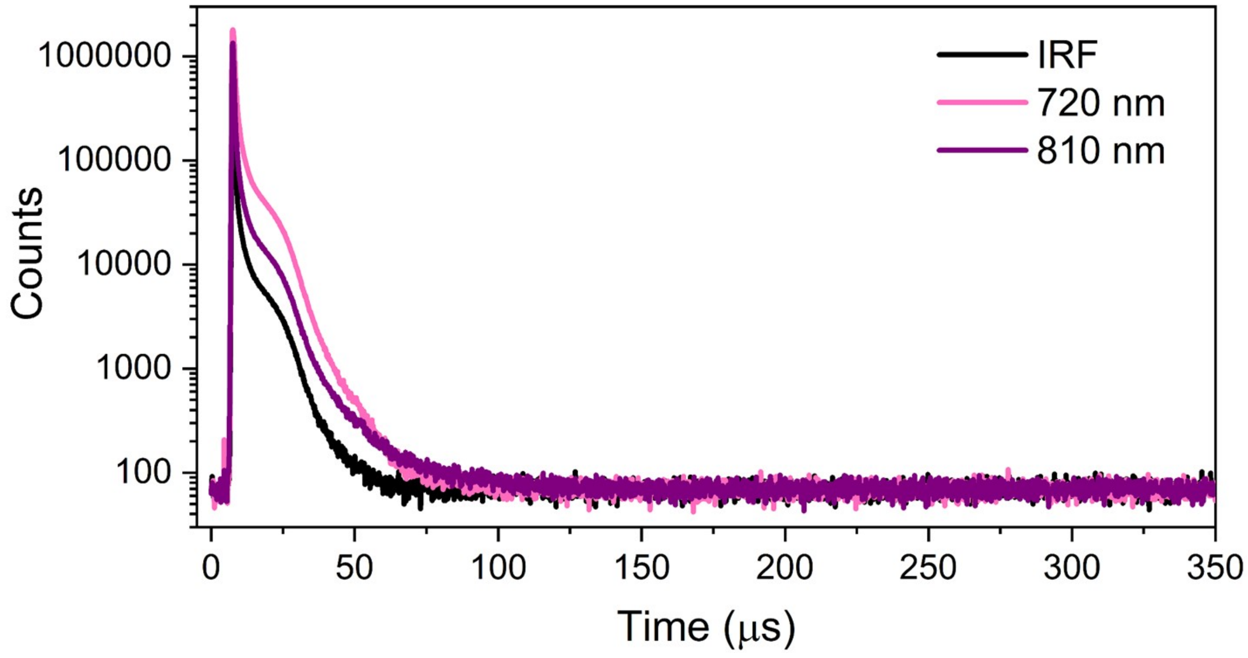

Figure S6. Decay curves of DNA-Ag_{16}NCs in a 10 mM NH_4OAc aqueous solution (HH condition) at 25 ^oC, exciting at 531 nm with a Xe flash lamp (repetition rate = 300 Hz). The black trace is the instrument response function, while pink and purple curves are the decays monitored at 720 and 810 nm, respectively.

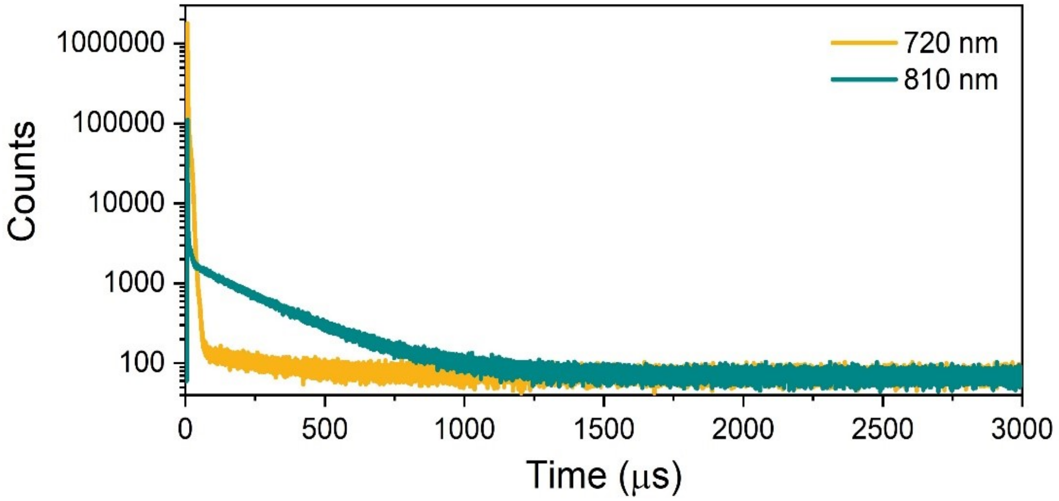

Figure S7. Decay curves of DNA-Ag_{16}NCs in a 10 mM NH_4OAc aqueous solution (HH condition) at -196 ^oC, exciting at 531 nm with a Xe flash lamp (repetition rate = 300 Hz). Yellow: luminescence monitored at λ_{em}=720 nm. Green: luminescence measured at λ_{em}=810 nm.

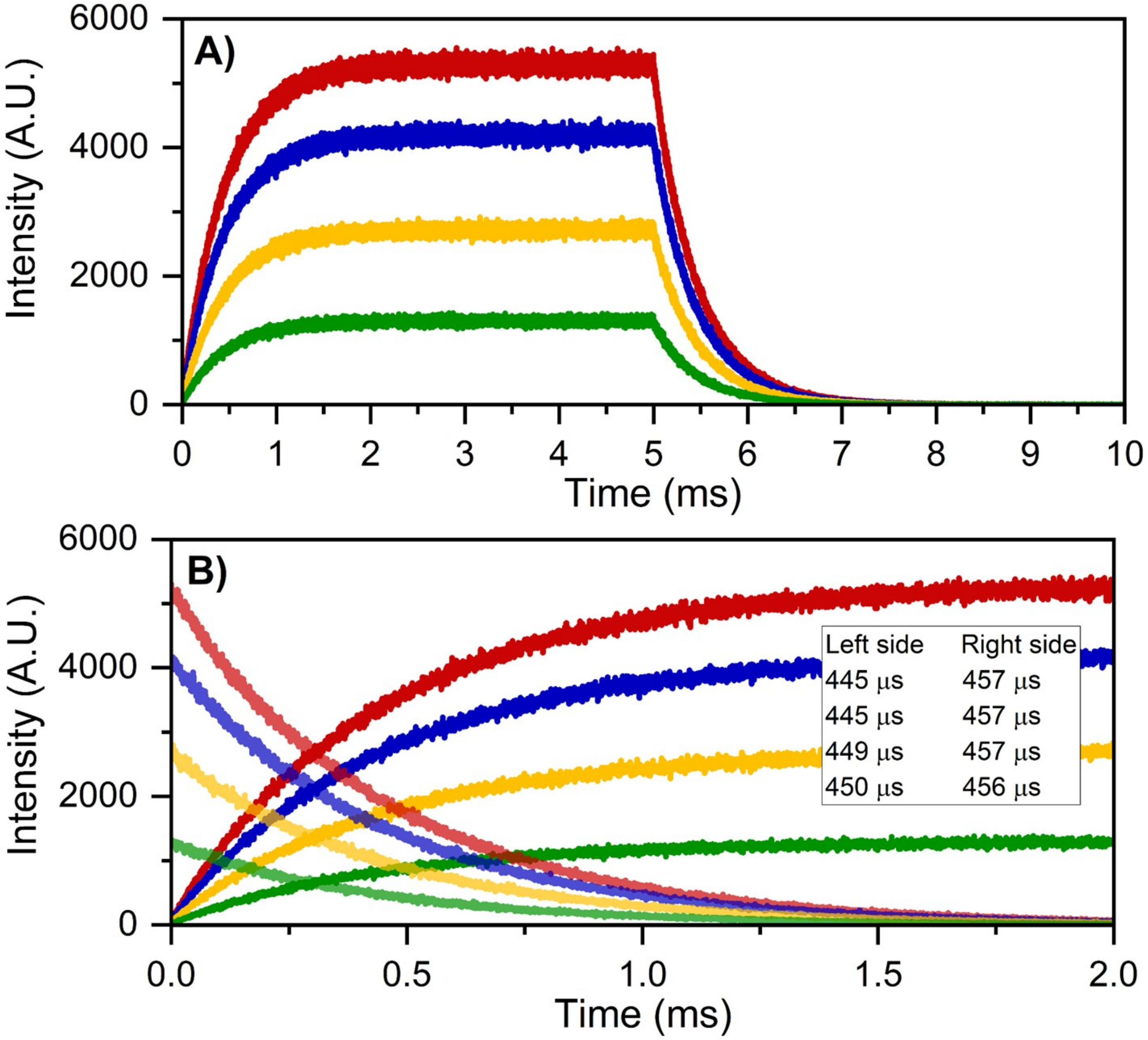

<u>Figure S8.</u> A) Emission of DNA-Ag_{16}NCs in the DD condition at -196 °C, recorded at 820 nm in burst mode, exciting at 507.5 nm. The laser power impinging on the sample was 86 µW (red), 65 µW (blue), 41 µW (yellow) and 14 µW (green), respectively. **B)** Zoom-in of the intensity build-up of the long-lived emission (right), and intensity decay upon switching off the laser (left). The time constants are reported on the graph. Details on the measurements can be found in paragraph 3.2.6B.

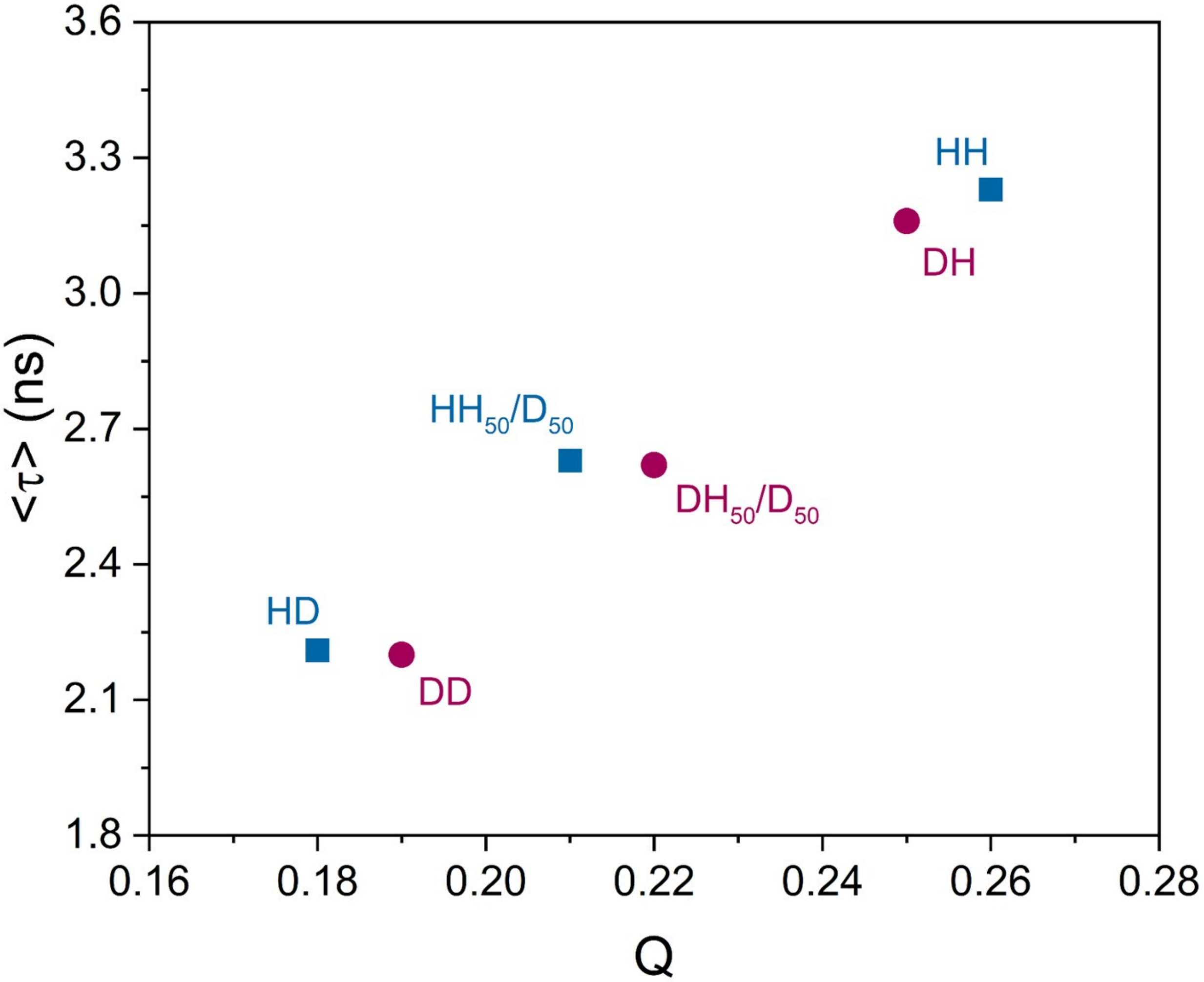

Figure S9. Intensity-weighted average decay time (<τ>) as a function of fluorescence quantum yield (Q) at 25 ºC for DNA-Ag_{16}NCs in different conditions. See Table S2 for details.

Solvent	Q	<τ> (ns)	Solvent	Q	<τ> (ns)
HH	0.26	3.23	DH	0.25	3.16
HH_{50}/D_{50}	0.21	2.63	DH_{50}/D_{50}	0.22	2.62
HD	0.18	2.21	DD	0.19	2.20

Table S2. Fluorescence quantum yield (Q) and intensity-weighted average decay time <τ> at 740 nm for DNA-Ag_{16}NCs synthesized and measured in different conditions at 25 ºC. <τ> values are also reported in Table 1.

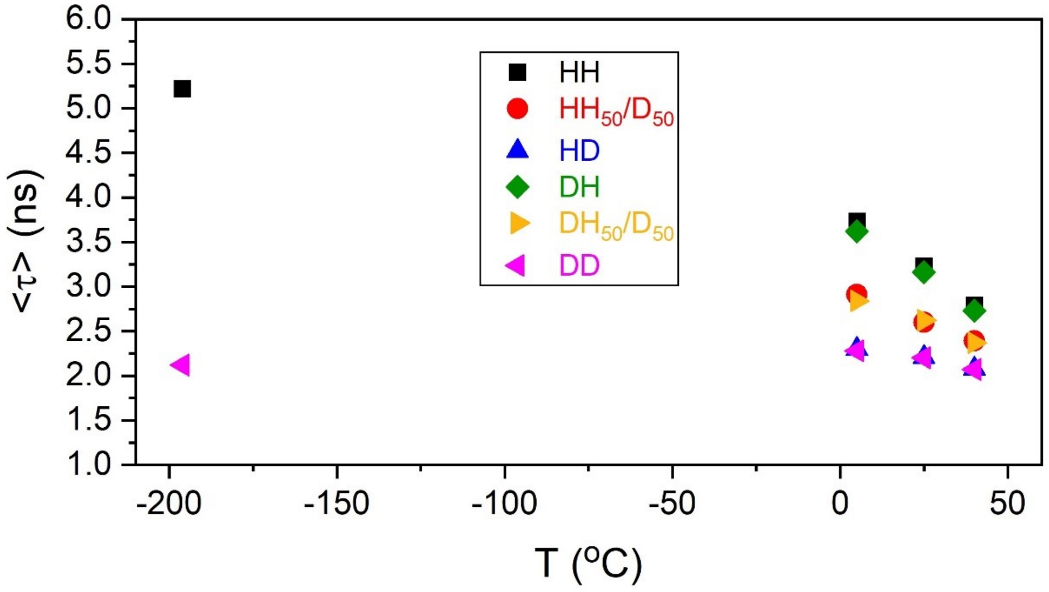

Figure S10. Intensity-weighted average decay time (<τ>) as a function of temperature for DNA-Ag_{16}NCs in different conditions. See Table 1 for details.

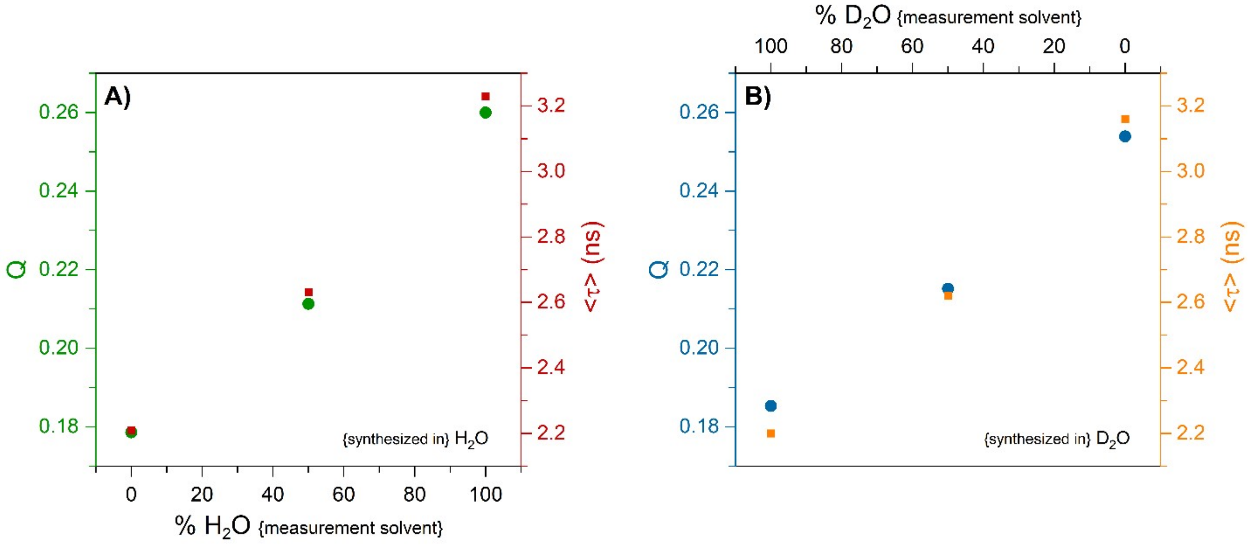

Figure S11. Fluorescence quantum yield (Q) and intensity-weighted average decay time (<τ>) at 25 °C for DNA-Ag_{16}NCs as a function of H_2O and D_2O content (%). DNA-Ag_{16}NCs were synthesized in **A)** 10 mM NH_4OAc H_2O solution, and **B)** 10 mM NH_4OAc D_2O solution. x-axes refer to the percentage of H_2O or D_2O in the measurement solvent.

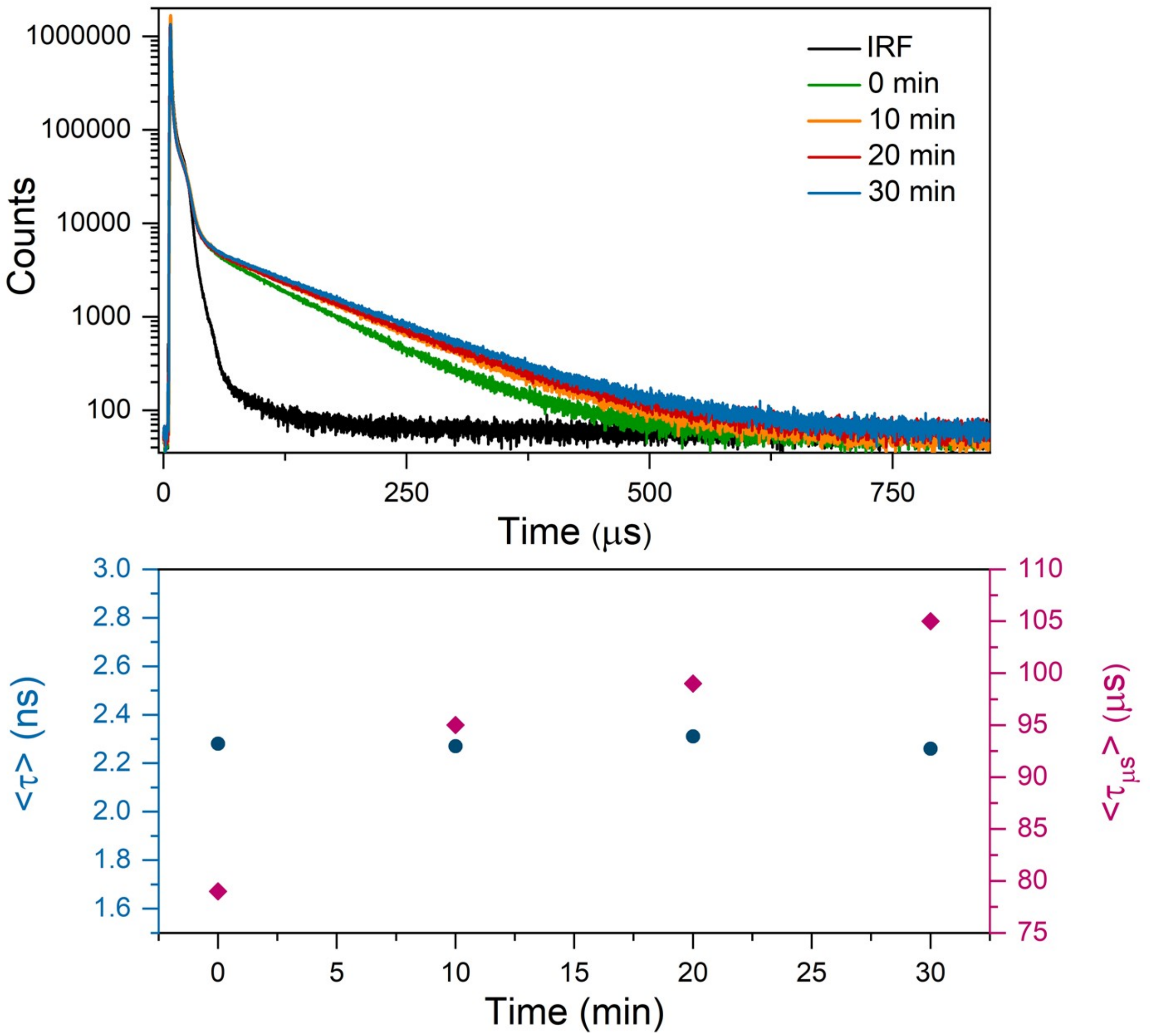

Figure S12. **A)** Microsecond decay curves of DNA-Ag_{16}NCs in the DD condition at 25 ºC, measured at 810 nm. The sample was excited at 510 nm with a Xe flash lamp (repetition rate = 300 Hz). The cuvette was connected to a water jet pump which lowered the head space pressure in the cuvette and hence decreased the amount of dissolved O_2 in the D_2O solution as a function of time. The µs-lived decay lengthens as a function of time (due to removal of dissolved O_2). **B)** <τ> and <$\tau_{\mu s}$> as a function of exposure time to the low pressure generated by the water jet pump. <τ> stays constant while <$\tau_{\mu s}$> clearly increases as a function of time.

DNA721-AgNC

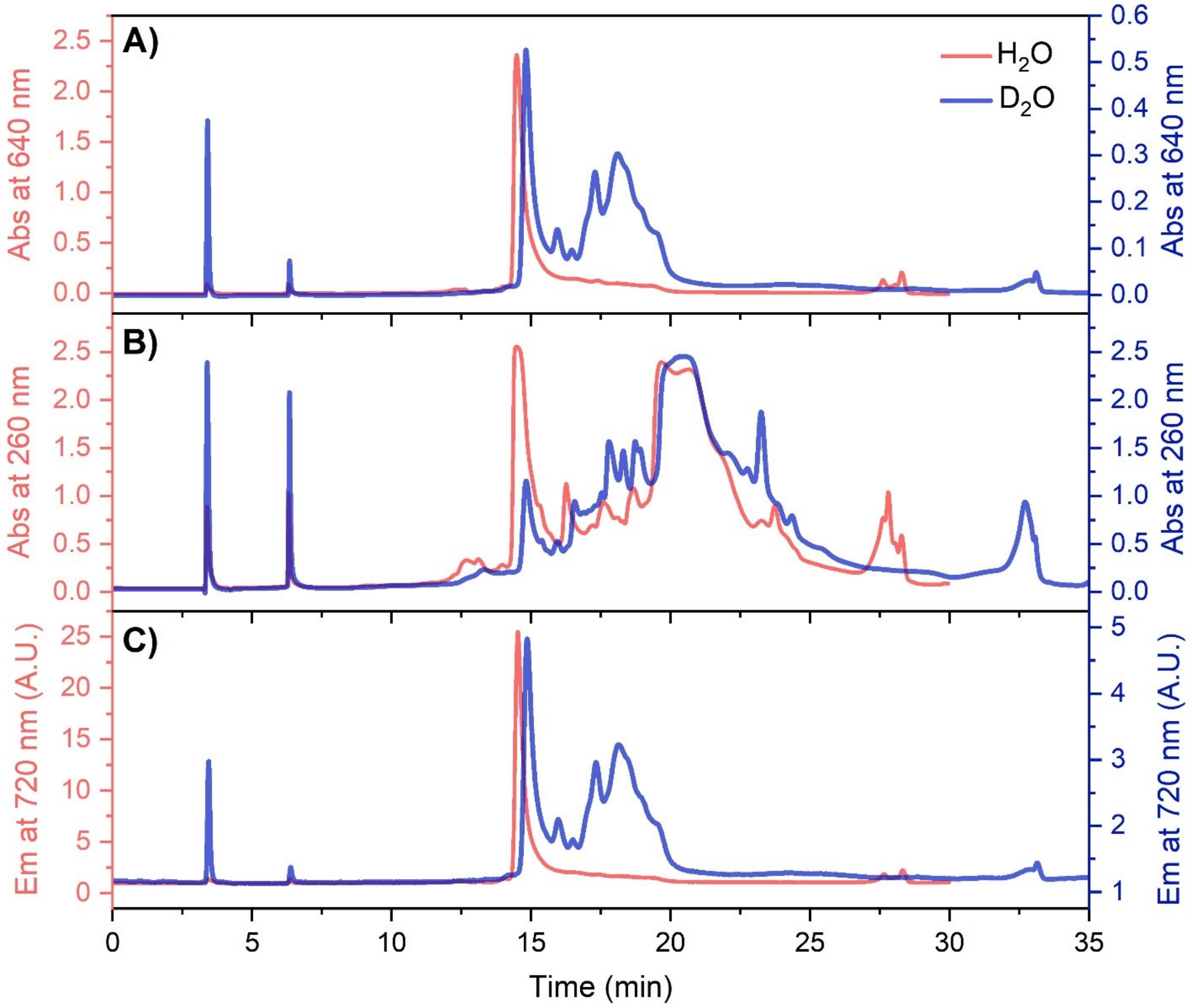

Figure S13. HPLC chromatograms of DNA721-AgNCs synthesized in 10 mM NH_4OAc H_2O (salmon) and D_2O (blue) solutions **A)** monitoring the main absorption peak of the clusters at 640 nm, and **B)** monitoring the DNA absorption at 260 nm. **C)** Chromatograms that monitor the emission of the clusters at 720 nm (λ_{exc}= 640 nm). The two HPLC methods are characterized by the same elution gradient until 22 min, but differ afterwards (see Table S1 for details). For the H_2O condition, the fraction was collected between 14 and 16 min, whereas 14.6-20 min was the collection interval for the sample prepared in D_2O.

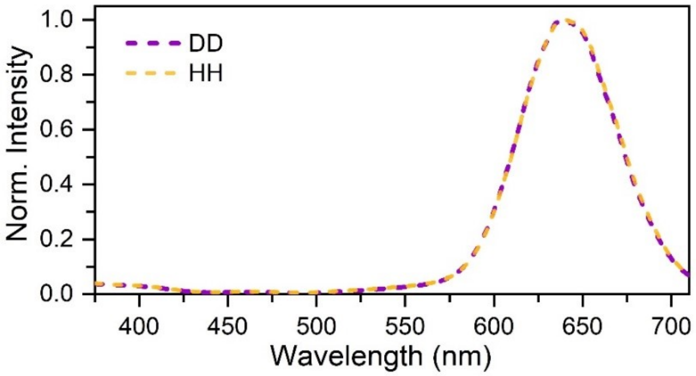

Figure S14. Normalized excitation spectra of DNA721-AgNCs in DD (purple) and HH (yellow) conditions at 25 °C, monitoring the emission at 720 nm.

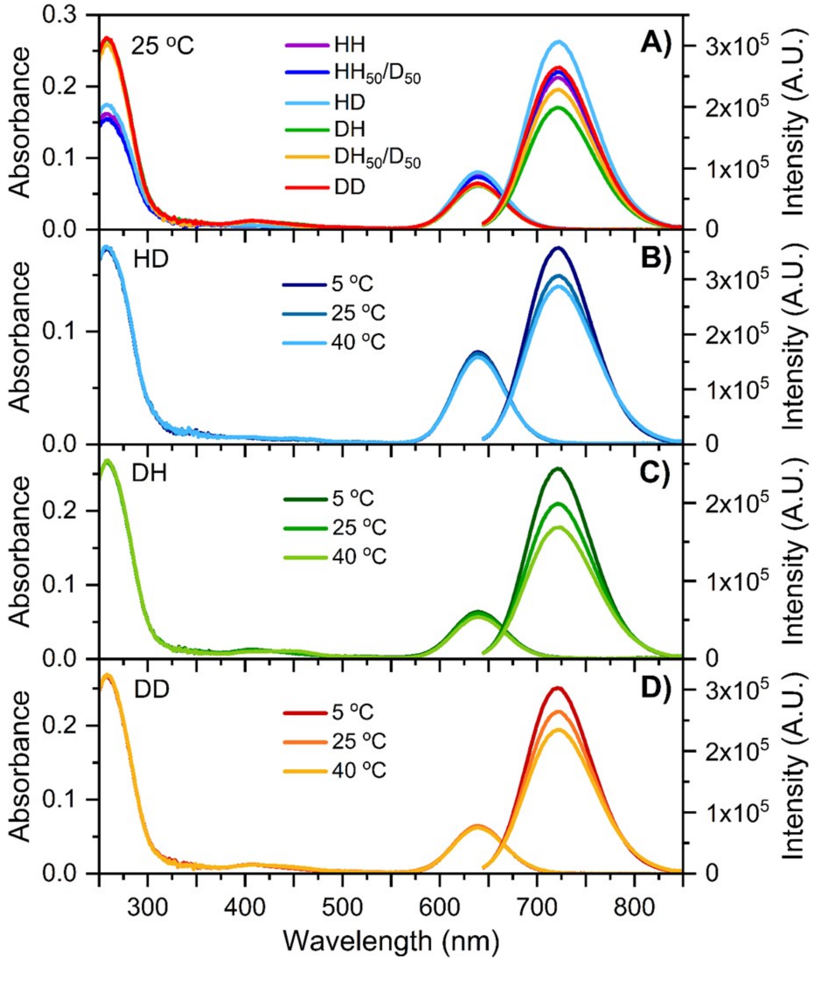

Figure S15. Absorption and emission spectra of DNA721-AgNCs in different conditions and temperatures used for fluorescence quantum yield (Q) calculations. **A)** Data measured at 25 °C. **B-D)** Data of DNA721-AgNCs in HD, DH and DD conditions at 5, 25, and 40 °C. The value of 0.73 for the HH condition at 25 °C was used as reference to calculate all other Q values.[1, 3]

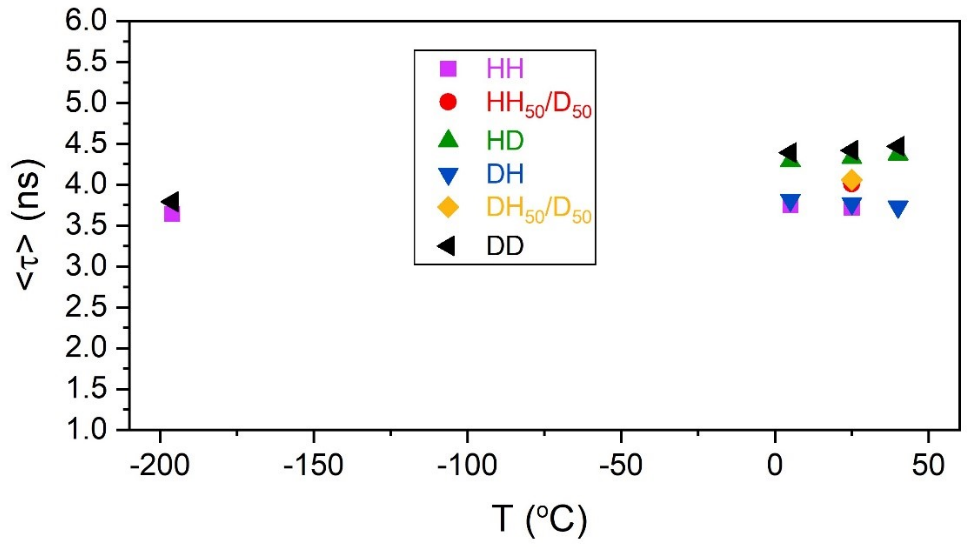

Figure S16. Intensity-weighted average decay time (<τ>) as a function of temperature for DNA721-AgNCs in different conditions. See Table 2 for details.

References.

1. A. M. Brouwer, *Pure and Applied Chemistry*, 2011, **83**, 2213.
2. S. A. Bogh, M. R. Carro-Temboury, C. Cerretani, S. M. Swasey, S. M. Copp, E. G. Gwinn and T. Vosch, *Methods and Applications in Fluorescence*, 2018, **6**, 024004.
3. V. A. Neacşu, C. Cerretani, M. B. Liisberg, S. M. Swasey, E. G. Gwinn, S. M. Copp and T. Vosch, *Chemical Communications*, 2020, **56**, 6384-6387.
4. J. R. Lakowicz, *Principles of fluorescence spectroscopy*, 2006.
5. J. T. Petty, S. Carnahan, D. Kim and D. Lewis, *The Journal of Chemical Physics*, 2021, **154**, 244302.
6. M. B. Liisberg, Z. Shakeri Kardar, S. M. Copp, C. Cerretani and T. Vosch, *The Journal of Physical Chemistry Letters*, 2021, **12**, 1150-1154.
